# Psychological Distress Is Associated with Emotional Overeating in Multiple Sclerosis: The Role of Perceived Disease Impact and Illness Acceptance—A Cross-Sectional Study

**DOI:** 10.3390/nu18162642

**Published:** 2026-08-12

**Authors:** Maciej Wilski, Jarosław Gabryelski, Waldemar Brola

**Affiliations:** 1 Department of Health and Rehabilitation Psychology, Poznań University of Physical Education, 61-871 Poznan, Poland; 2 Division of Rehabilitation Engineering, Institute of Transport, Poznan University of Technology, 61-131 Poznan, Poland; jaroslaw.gabryelski@put.poznan.pl; 3 Collegium Medicum, Jan Kochanowski University, 25-269 Kielce, Poland; wbrola@wp.pl

**Keywords:** multiple sclerosis, emotional overeating, psychological distress, illness acceptance, eating behaviour, nutritional psychiatry

## Abstract

**Background/Objectives:** Emotional overeating may be relevant to nutritional self-management in people with multiple sclerosis (MS), but its links with disease-related appraisals and psychological adjustment remain unclear. This study examined associations between perceived physical impact of MS and emotional overeating, considering illness acceptance and psychological distress. **Methods:** This secondary cross-sectional study included 382 adults with MS recruited from specialist clinical centres in Poland. Emotional overeating, perceived physical impact, illness acceptance, and psychological distress were assessed using the My Eating Habits Questionnaire, Multiple Sclerosis Impact Scale-29 physical subscale, Acceptance of Illness Scale, and 12-item General Health Questionnaire. Hierarchical regression and serial indirect-association models were adjusted for disease duration and Expanded Disability Status Scale score. Indirect associations were evaluated using 5000 bootstrap resamples. **Results:** Greater perceived physical impact was associated with emotional overeating at the bivariate level. In the adjusted regression model, greater psychological distress was associated with higher emotional overeating (B = 0.169, β = 0.312, *p* < 0.001); the model explained 20.1% of outcome variance. The total association between physical impact and emotional overeating was statistically significant (B = 0.038, *p* < 0.001), whereas the direct association was not (B = 0.010, *p* = 0.216). Indirect associations were supported through psychological distress alone (estimate = 0.016, 95% CI 0.009–0.025) and sequentially through lower illness acceptance and greater psychological distress (estimate = 0.006, 95% CI 0.003–0.009). **Conclusions:** Emotional overeating was more closely associated with psychological distress and illness adjustment than with perceived physical disease impact alone. Nutritional care may benefit from attention to emotional triggers for eating and psychological distress; longitudinal studies are needed to establish temporal relationships.

## 1. Introduction

Dietary behaviours are a potentially modifiable component of health management in chronic conditions. In people with multiple sclerosis (MS), nutrition and diet-related behaviours are frequently considered within the broader context of self-management and patient education [1,2]. Among eating-related behaviours, emotional eating has received considerable attention in nutrition and behavioural research. Emotional eating is commonly defined as the tendency to eat in response to emotional cues rather than internal physiological signals of hunger and satiety [3]. Although the construct has traditionally been linked to overeating in response to negative affect, both negative and positive emotional states may influence eating behaviour, and the direction of this response may vary across individuals and situations [3,4].

Emotional eating is relevant from a nutritional perspective because it has been associated with less favourable dietary patterns, including a greater preference for energy-dense and highly palatable foods, as well as with overweight or obesity, depressive symptoms, anxiety, and perceived stress [5,6]. Food may serve as a readily available means of regulating unpleasant affective states; however, the mechanisms underlying emotional eating are heterogeneous and cannot be reduced to a single process [3,7]. Moreover, much of the available evidence is cross-sectional, which limits conclusions regarding the temporal ordering of emotional distress and eating behaviour.

MS is a chronic, immune-mediated disease of the central nervous system that may involve fatigue, pain, sensory symptoms, mobility limitations, cognitive difficulties, and restrictions in everyday functioning [8]. Dietary issues are of substantial interest to many people with MS, and disease-specific diets are commonly discussed and used despite persisting uncertainty regarding their effectiveness for disease-related outcomes [2]. Previous research has therefore examined dietary behaviours, nutritional beliefs, and diet-focused education in this population [1,2]. In contrast, emotional eating has rarely been examined in people with MS, particularly within an integrated model that combines perceived disease impact, illness acceptance, and current psychological distress. This represents an important gap because these variables may jointly reflect how individuals appraise, accommodate, and emotionally respond to the burden of living with MS.

The psychological consequences of MS are shaped not only by objectively assessed neurological disability but also by the individual’s appraisal of illness-related symptoms, limitations, and uncertainty [9]. Perceived physical impact of MS reflects the subjective experience of how the disease affects physical functioning and everyday activities. Although perceived disease impact may be related to neurological disability, it is not equivalent to disability assessed clinically. Previous research has shown that perceived impact of MS is associated with coping processes and self-management-related outcomes, indicating that subjective illness appraisals may be relevant to health-related behaviours [10].

Illness acceptance may represent another important aspect of adjustment to MS. It refers to the extent to which an individual accommodates illness-related limitations within everyday life, rather than remaining in persistent conflict with circumstances that cannot be fully controlled. Greater acceptance of MS has been associated with more favourable psychological adjustment [11]. In addition, illness acceptance has been related to better health-related quality of life among people with MS, alongside coping strategies and disease severity [12]. Difficulties in accepting illness-related limitations may therefore co-occur with greater emotional burden and a stronger tendency to rely on immediately available strategies of affect regulation.

Psychological distress is common in people with MS and may adversely affect everyday functioning, quality of life, and engagement in health-related behaviours. A systematic review and meta-analysis indicated that depression and anxiety affect a substantial proportion of people with MS [13]. Previous research has further shown that mental health in MS is associated with psychological variables and coping processes [10]. In non-MS populations, depressive symptoms, anxiety, and perceived stress have been consistently associated with emotional eating [5,6]. Thus, psychological distress may be particularly relevant to understanding emotional eating among individuals living with a chronic and unpredictable neurological condition.

Taken together, perceived physical impact of MS, illness acceptance, psychological distress, and emotional overeating may represent interconnected aspects of psychological and behavioural adjustment. Illness acceptance was positioned before psychological distress because it reflects a broad illness-related appraisal of enduring limitations, reduced independence, and reliance on others, whereas psychological distress reflects a more proximal state of current emotional strain. Accordingly, lower illness acceptance was conceptualised as an adjustment-related factor that may be statistically linked with greater current distress. This ordering was used as a theory-informed decomposition of cross-sectional associations and should not be interpreted as evidence that changes in illness acceptance temporally precede changes in psychological distress. Alternative and reciprocal associations remain plausible.

Therefore, the present secondary analysis addressed this gap by examining associations between perceived physical impact of MS and emotional overeating, considering illness acceptance and psychological distress as serially ordered variables. Disease duration and neurological disability, assessed using the Expanded Disability Status Scale (EDSS), were included as covariates. We hypothesised that greater perceived physical impact of MS would be associated with lower illness acceptance, greater psychological distress, and higher emotional overeating. Given the cross-sectional design, all findings were interpreted as associations.

## 2. Materials and Methods

### 2.1. Study Design and Setting

This study was a secondary cross-sectional analysis of data collected as part of a broader questionnaire-based research project examining psychosocial functioning among people with multiple sclerosis. The parent study was conducted in cooperation with two specialist clinical centres in Poland: the Multiple Sclerosis Rehabilitation Center in Borne Sulinowo and the Department of Neurology at the Specialist Hospital in Końskie. Data were collected between December 2015 and January 2017. The present analysis used the complete-case dataset and focused on emotional overeating, perceived physical impact of MS, illness acceptance, and psychological distress.

### 2.2. Participants and Procedure

The study included a convenience sample of 382 adults with multiple sclerosis (MS), including 256 women and 126 men. Participants were recruited during routine control visits at the collaborating centres. The sample was not recruited consecutively; rather, eligible patients who were available during the recruitment period and met the study criteria were invited to participate.

Potential participants were screened by a member of the research team before invitation to participate. Eligibility was determined on the basis of available clinical records and routine clinical assessment. Inclusion criteria were: (1) neurologist-confirmed diagnosis of definite MS according to the revised McDonald criteria [14]; (2) no clinical relapse during the 30 days preceding enrolment; and (3) ability to provide written informed consent and complete the self-report questionnaire independently.

Individuals with a documented psychotic disorder or clinically apparent cognitive impairment were not invited when the treating neurologist or research staff considered that these conditions could compromise informed consent or reliable questionnaire completion. No formal neuropsychological screening instrument was administered specifically for study eligibility; therefore, the possible influence of mild or subclinical cognitive difficulties on questionnaire responses cannot be fully excluded. Co-occurring somatic conditions were not blanket exclusion criteria unless they were judged likely to preclude questionnaire completion or substantially affect participation.

Eligible individuals received verbal information regarding the study aims and procedures and were invited to participate during routine visits at the collaborating centres. Those who agreed to participate provided written informed consent and completed a confidential self-report questionnaire individually in a quiet room designated for the study. Questionnaires were checked for completeness immediately after they were returned, and respondents were invited to complete any apparent unintentional omissions whenever possible. Of the 400 patients initially selected for the study, 18 were excluded from the final analytical sample because their questionnaires contained incomplete data. No statistical imputation was performed. The final analytical dataset therefore comprised 382 participants with complete data for all variables included in the analyses.

This non-interventional study was based exclusively on questionnaire data and did not involve any experimental intervention, modification of routine clinical care, additional diagnostic or monitoring procedures, or collection of biological material. In accordance with the institutional procedures in place at the time of data collection, the project was classified as a non-interventional questionnaire-based study and was therefore not subject to formal ethical review by the Bioethics Committee at Poznan University of Medical Sciences. The study was conducted in accordance with the principles of the Declaration of Helsinki and was reported in accordance with the Strengthening the Reporting of Observational Studies in Epidemiology (STROBE) guidelines for cross-sectional studies.

### 2.3. Measures

#### 2.3.1. Emotional Overeating

Emotional overeating was assessed using Factor II of the My Eating Habits Questionnaire (MEH; Moje Zwyczaje Żywieniowe) developed by Ogińska-Bulik and Putyński [15]. The MEH is an original Polish self-report questionnaire designed to assess selected eating habits in adults and older adolescents.

The questionnaire comprises 30 statements answered using a dichotomous “yes” or “no” format. It includes three 10-item dimensions: Factor I—habitual overeating, Factor II—emotional overeating, and Factor III—restrictive dieting. For items 2, 5, 13, 17, and 23, a “no” response was assigned 1 point; for all remaining items, a “yes” response was assigned 1 point. Scores for each dimension range from 0 to 10, with higher scores indicating a stronger tendency towards the respective eating pattern.

Only Factor II—emotional overeating—was used in the present study. This 10-item subscale assesses a self-reported tendency to consume food in response to emotional states, including being upset or angry, mood-dependent eating, and difficulties stopping eating. The MEH has also been used in recent nutrition-related studies conducted in Polish samples [16,17]. Although the MEH was not developed specifically for people with MS, it was selected because it is a Polish-language instrument assessing eating patterns that correspond directly to the construct of emotional overeating examined in the present study. In this sample, the emotional overeating subscale demonstrated satisfactory internal consistency (Cronbach’s α = 0.80). The score was therefore analysed as a dimensional self-report indicator of emotional overeating rather than as a diagnostic measure of binge-eating disorder or other eating disorders.

#### 2.3.2. Perceived Physical Impact of Multiple Sclerosis

Perceived physical impact of multiple sclerosis (MS) was assessed using the physical subscale of the Polish adaptation of the Multiple Sclerosis Impact Scale-29 (MSIS-29) [18,19]. The MSIS-29 is a well-established, disease-specific patient-reported outcome measure designed to assess the perceived physical and psychological impact of MS during the preceding 2 weeks [18,20].

In the present study, the 20-item physical subscale was used. The items assess the functional impact of MS on everyday life and are rated on a five-point response scale. Raw scores range from 20 to 100, with higher scores indicating a greater perceived physical impact of the disease. The Polish adaptation demonstrated satisfactory psychometric properties and showed meaningful associations with neurological disability, fatigue, depressive symptoms, and MS-specific quality of life [19]. The physical MSIS-29 subscale has also demonstrated satisfactory validity and reliability in studies involving people with MS [20,21] and has been used previously in research involving Polish people with MS [12]. Internal consistency of the physical subscale in the present study is reported in Section 3.

#### 2.3.3. Illness Acceptance

Illness acceptance was assessed using the Acceptance of Illness Scale (AIS), originally developed by Felton, Revenson, and Hinrichsen [22] and adapted for use in Poland by Juczyński [23]. The AIS is a generic self-report measure of psychological adjustment to chronic illness. It assesses the extent to which an individual accommodates illness-related consequences and limitations in everyday life.

The Polish adaptation comprises eight statements addressing negative consequences of illness, including limitations imposed by the disease, reduced independence, reliance on other people, and diminished self-esteem. Participants rate each statement on a five-point Likert-type scale, ranging from 1 (“strongly agree”) to 5 (“strongly disagree”). The total score ranges from 8 to 40, with higher scores indicating greater acceptance of illness and better adjustment to illness-related limitations.

The Polish adaptation has demonstrated satisfactory psychometric properties in studies of adults with chronic health conditions [23], and the AIS has been used previously in research involving people with MS [24]. Internal consistency of the AIS in the present sample is reported in Section 3.

#### 2.3.4. Psychological Distress

Current psychological distress was assessed using the Polish adaptation of the 12-item General Health Questionnaire (GHQ-12), developed by Goldberg and Williams [25] and adapted for use in Poland by Makowska and Merecz [26]. The GHQ-12 is a brief self-report screening measure of non-psychotic psychological distress in adults and was designed for use in community samples and non-psychiatric clinical settings.

The questionnaire includes 12 items referring to recent changes in concentration, sleep, perceived strain, decision-making, ability to cope with difficulties, everyday functioning, mood, and self-confidence. Each item is rated on a four-point response scale reflecting whether the respondent experienced a given state less than usual, no more than usual, rather more than usual, or much more than usual during the preceding few weeks.

For the present analyses, responses were coded from 1 to 4 and summed after reverse-coding positively worded items. Total scores could therefore range from 12 to 48, with higher scores indicating greater current psychological distress. The GHQ-12 total score was analysed as a unidimensional indicator of psychological distress rather than as a diagnostic measure of a specific psychiatric disorder [27].

The Polish adaptation has demonstrated satisfactory validity for identifying psychological distress in adult populations [26]. The GHQ has also been used in studies involving people with MS, including research supporting its sensitivity for detecting emotional disturbance in this population [28,29]. Internal consistency of the scale in the present study is reported in Section 3.

#### 2.3.5. Clinical Characteristics

Demographic and clinical information were collected using a standardized questionnaire. For the purposes of the present study, disease duration was expressed as the number of years since the diagnosis of multiple sclerosis.

Neurological disability was assessed by a neurologist using the Expanded Disability Status Scale (EDSS) [30]. The EDSS is a clinician-administered measure of neurological impairment and disability in multiple sclerosis that is based on the neurological examination and assessment of functional systems. It remains one of the most widely used disability outcome measures in MS research and clinical trials [30,31].

EDSS scores range from 0, indicating a normal neurological examination, to 10, indicating death due to MS, with higher scores reflecting greater neurological disability. Disease duration and EDSS score were included as continuous covariates in the statistical analyses because both variables may be related to functioning and health-related outcomes in people with MS. Although the EDSS is an ordinal clinician-rated scale, it was entered as a continuous covariate because it was used for parsimonious adjustment rather than as the primary exposure, and this approach retained information across the broad observed range of scores in the present sample (1.0–8.5).

### 2.4. Statistical Analysis

Statistical analyses were performed using IBM SPSS Statistics for Windows, version 29.0 (IBM Corp., Armonk, NY, USA). Serial indirect associations were estimated using PROCESS macro version 4.2 (Model 6) with 5000 percentile bootstrap resamples.

Because the present study was a secondary analysis of an existing dataset, no software-based a priori sample size calculation was performed specifically for the current model, and no expected effect size, statistical power, or significance level was specified in advance for this secondary analysis. The analytical sample size was determined by the number of participants from the parent study with complete data on all variables included in the present analyses. The final sample of 382 participants exceeded common recommendations for multiple regression models with five predictors and provided sufficient precision for estimating the planned regression-based indirect associations.

Descriptive statistics were calculated for all study variables. Continuous variables are presented as means and standard deviations, together with observed ranges where appropriate, whereas categorical variables are presented as frequencies and percentages. Distributional characteristics were evaluated using the Shapiro–Wilk test and visual inspection of histograms and normal Q–Q plots.

Internal consistency of the multi-item measures was assessed using Cronbach’s alpha coefficients. Pearson product–moment correlation coefficients were calculated to examine bivariate associations among emotional overeating, perceived physical impact of MS, illness acceptance, psychological distress, disease duration, and EDSS score.

Hierarchical multiple linear regression analysis was conducted to examine independent associations with emotional overeating. The MEH Factor II score, ranging from 0 to 10, was analysed as an approximately continuous outcome because it represents a summed score across 10 dichotomous items. This decision was further supported by visual inspection of residual plots and the absence of meaningful violations of linear-regression assumptions in the final model. Disease duration and EDSS score were entered in Step 1, perceived physical impact of MS in Step 2, illness acceptance in Step 3, and psychological distress in Step 4. For each step, the proportion of explained variance, adjusted explained variance, change in explained variance, and corresponding F statistics were reported. Unstandardised regression coefficients, standard errors, standardised regression coefficients, 95% confidence intervals, and two-sided *p*-values are reported for the final model.

A regression-based serial indirect-association model, following the approach described by Hayes [32], was estimated with perceived physical impact of MS as the independent variable, emotional overeating as the dependent variable, and illness acceptance and psychological distress entered as serially ordered variables. The order was specified a priori because illness acceptance represents an illness-related adjustment appraisal, whereas the GHQ-12 captures current psychological distress. This order was used solely to parameterise a theory-informed decomposition of cross-sectional associations and was not interpreted as evidence of temporal or causal precedence. Disease duration and EDSS score were included as covariates in the total-association model and in all constituent regression equations.

Standardized path coefficients reported in the Section 3 were obtained from separately fitted ordinary least-squares regression equations using z-standardized variables. These regression equations corresponded to the constituent equations of the serial indirect-association model and included the same covariates as the PROCESS analysis. Unstandardised coefficients and bootstrap confidence intervals from the PROCESS analysis are reported in Section 3.

Indirect associations were estimated for the pathways through illness acceptance alone, psychological distress alone, and the sequential pathway through illness acceptance and psychological distress. Indirect-association estimates and their 95% confidence intervals were obtained using 5000 percentile bootstrap resamples. An indirect association was regarded as statistically supported when the corresponding 95% bootstrap confidence interval did not include zero. Given the cross-sectional design, the serial model was interpreted as a theoretically informed pattern of statistical associations rather than evidence of temporal ordering or causality.

Sensitivity analyses were performed to assess the robustness of the primary findings. First, age and sex were added as additional covariates to all regression equations in the serial indirect-association model. Second, the total MSIS-29 score was substituted for the physical impact subscale. The same bootstrap procedure was used in both sensitivity analyses.

Multicollinearity was assessed using variance inflation factors. Influential observations were evaluated using Cook’s distance, and the Durbin–Watson statistic was calculated to assess residual autocorrelation. All tests were two-sided, and *p*-values < 0.05 were considered statistically significant.

## 3. Results

### 3.1. Participant Characteristics

Of the 400 patients initially selected for the study, 18 were excluded because of incomplete questionnaire data. The final analytical sample comprised 382 people with multiple sclerosis (MS), including 256 women (67.0%) and 126 men (33.0%). Participants had a mean age of 46.4 years (SD = 11.9; range: 18–82 years). Most participants were married (67.3%), and the largest educational category was secondary education (43.2%), followed by higher education (31.7%). Nearly half of the participants were receiving a disability pension (47.9%), while 38.2% were employed. The mean disease duration was 11.5 years (SD = 9.1; range: 1–48 years), and the mean EDSS score was 4.35 (SD = 1.68; range: 1.0–8.5). Relapsing–remitting MS was the most common disease phenotype (41.4%), followed by primary progressive MS (23.0%), secondary progressive MS (18.3%), and progressive–relapsing MS (8.6%). Information on MS phenotype was unavailable for 33 participants (8.6%). Selected sociodemographic and clinical characteristics are presented in Table 1.

### 3.2. Descriptive Statistics, Reliability, and Bivariate Associations

Descriptive statistics and internal consistency coefficients are presented in Table 2, and Pearson correlations are presented in Table 3. Emotional overeating scores ranged from 0 to 10, with a mean of 4.03 (SD = 2.78). All multi-item measures demonstrated satisfactory to excellent internal consistency (Cronbach’s α = 0.80–0.98).

Greater perceived physical impact of MS was positively associated with emotional overeating, whereas illness acceptance was negatively associated with emotional overeating. Psychological distress showed the strongest positive correlation with emotional overeating. Both disease duration and EDSS score were negatively associated with emotional overeating.

Greater perceived physical impact of MS was also associated with lower illness acceptance and more psychological distress. Illness acceptance was inversely associated with psychological distress. Disease duration was positively associated with EDSS score and negatively associated with illness acceptance.

### 3.3. Hierarchical Regression Analysis Predicting Emotional Overeating

The hierarchical regression analysis is presented in Table 4. Disease duration and EDSS score entered in the first step explained 4.8% of the variance in emotional overeating. Adding perceived physical impact of MS in the second step explained an additional 6.4% of the variance. Illness acceptance contributed a further 2.1%, whereas psychological distress accounted for an additional 6.9% of the variance.

The final model was statistically significant, F(5, 376) = 18.93, *p* < 0.001, and explained 20.1% of the variance in emotional overeating. Greater psychological distress was independently associated with higher emotional overeating. In contrast, longer disease duration and higher EDSS scores were independently associated with lower emotional overeating. Perceived physical impact of MS and illness acceptance were not independently associated with emotional overeating after psychological distress was included in the model.

Regression diagnostics indicated no meaningful multicollinearity (VIF range = 1.35–1.50). Visual inspection of standardized residual-versus-predicted-value plots indicated no material departures from linearity or homoscedasticity, and normal Q–Q plots did not suggest substantial departures from normality. No single observation exerted undue influence on the model (maximum Cook’s distance = 0.034; Table S1).

### 3.4. Serial Indirect Associations Between Perceived Physical Impact of MS and Emotional Overeating

The results of the serial indirect-association model are presented in Table 5. After adjustment for disease duration and EDSS score, greater perceived physical impact of MS was associated with lower illness acceptance and greater psychological distress. Lower illness acceptance was also associated with greater psychological distress. In the final equation, psychological distress was positively associated with emotional overeating, whereas the direct association between perceived physical impact of MS and emotional overeating was not statistically significant.

The total association between perceived physical impact of MS and emotional overeating was statistically significant (B = 0.038, *p* < 0.001). However, the direct association was not statistically significant after illness acceptance and psychological distress were included in the model (B = 0.010, *p* = 0.216).

**Figure 1:**
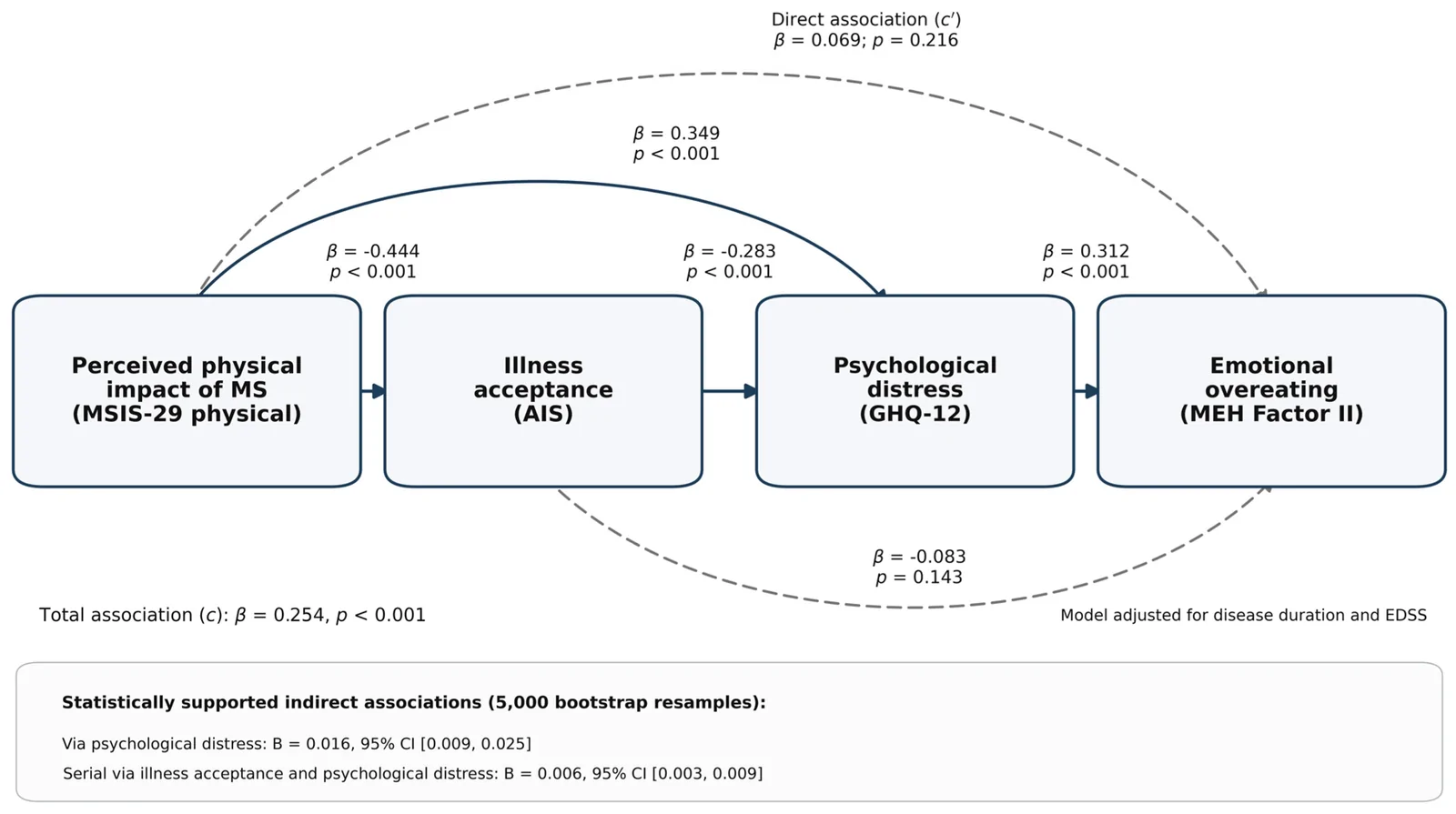
Adjusted serial indirect-association model linking perceived physical impact of multiple sclerosis with emotional overeating through illness acceptance and psychological distress. Standardized regression coefficients, derived from separately fitted regression equations using z-standardized variables, are shown. Disease duration and Expanded Disability Status Scale score were included as covariates in all regression equations. Solid arrows indicate statistically significant associations; dashed arrows indicate non-significant associations.

Bootstrap analyses indicated a statistically supported indirect association through psychological distress alone (estimate = 0.016, 95% CI 0.009–0.025). The sequential indirect association through lower illness acceptance and greater psychological distress was also statistically supported (estimate = 0.006, 95% CI 0.003–0.009). The indirect association through illness acceptance alone was not statistically supported. The total indirect association was statistically supported (estimate = 0.028, 95% CI 0.017–0.039). The adjusted serial indirect-association model is illustrated in Figure 1.

### 3.5. Sensitivity Analyses

The pattern of indirect associations remained unchanged after additional adjustment for age and sex. The total indirect association remained statistically supported (estimate = 0.028, 95% CI 0.017–0.040), as did the sequential indirect association through illness acceptance and psychological distress (estimate = 0.006, 95% CI 0.003–0.009).

In an alternative model in which the total MSIS-29 score replaced the physical impact subscale, the total indirect association remained statistically supported (estimate = 0.018, 95% CI 0.010–0.027). The sequential indirect association through illness acceptance and psychological distress was also statistically supported (estimate = 0.003, 95% CI 0.001–0.005). Detailed results are provided in Table S2.

## 4. Discussion

The present secondary analysis examined emotional overeating in people with multiple sclerosis (MS) within a psychosocial model incorporating perceived physical disease impact, illness acceptance, and psychological distress. Three findings warrant particular attention. First, greater perceived physical impact of MS was associated with higher emotional overeating at the bivariate level. Second, this association was no longer statistically significant after illness acceptance and psychological distress were considered simultaneously. Third, psychological distress was the strongest independent correlate of emotional overeating, and the indirect associations through psychological distress alone and sequentially through lower illness acceptance and greater psychological distress were statistically supported. Taken together, these results suggest that emotional overeating in people with MS may be embedded more closely in psychological adjustment to the disease than in the perceived physical impact of MS alone. Because the study was cross-sectional, the observed serial pattern should be interpreted as a theoretically informed pattern of associations rather than evidence of temporal ordering or causality.

The amount of explained variance should also be interpreted in this context. The final regression model explained approximately one-fifth of the variance in emotional overeating, indicating that the variables included in the model—particularly psychological distress—are relevant but not exhaustive correlates of this behaviour. Emotional overeating in MS is likely to be shaped by additional biological, behavioural, clinical, and social factors not included in the present model, such as fatigue, sleep disturbances, pain, appetite changes, medication use, physical activity, social support, and the broader dietary environment.

Research specifically addressing eating-related difficulties in MS remains limited and methodologically heterogeneous. Most previous studies have focused on broad eating attitudes, possible eating disorders, dietary quality, or adherence to dietary patterns rather than emotional overeating as a distinct behavioural tendency. For example, Kara and Çelik [33] found that higher scores on the Eating Attitudes Test were associated with poorer physical and mental health-related quality of life in a small sample of people with relapsing–remitting MS. Similarly, Saadat and Hosseininezhad [34] reported differences in emotional, external, and restrained eating between people with MS who had and had not experienced relapse during the preceding year. However, neither study can determine whether eating-related difficulties preceded, followed, or co-occurred with poorer health status or relapse history. The present findings add to this small body of literature by examining emotional overeating together with disease-related appraisals and psychological adjustment variables that may be particularly relevant in MS. These comparisons also suggest that discrepancies across studies may partly reflect differences in the eating-related constructs assessed, because emotional overeating, restrained eating, external eating, broad eating attitudes, and eating disorder risk are related but not interchangeable outcomes.

The central role of psychological distress in the present model is consistent with broader evidence on psychological functioning in MS. Dehghan et al. [35] reported that difficulties in emotion regulation were positively associated with psychological distress and negatively associated with quality of life in people with MS. In their model, lower MS self-efficacy was related to greater emotion-regulation difficulties and distress. Although illness acceptance and self-efficacy represent different psychological constructs, both may reflect the extent to which a person perceives that they can accommodate disease-related demands and maintain a sense of agency despite illness-related limitations. Thus, the present findings point to psychological distress as the most proximal correlate of emotional overeating in the model, whereas perceived physical impact and illness acceptance appear to be linked with emotional overeating mainly through their associations with current distress.

The association between psychological distress and emotional overeating is also compatible with previous evidence linking eating-related difficulties with poorer psychological functioning in MS. In the study by Kara and Çelik [33], higher risk of eating disorder was associated with lower mental health-related quality of life and greater role limitations related to emotional problems. However, the Eating Attitudes Test primarily captures eating attitudes and risk of eating disorders, whereas the emotional overeating subscale used in the present study assesses a tendency to eat excessively in response to emotional states. These constructs overlap only partially. Emotional overeating should therefore not be equated with a clinical eating disorder. Nevertheless, both findings support the relevance of psychological and emotional factors for eating-related behaviours in MS.

Illness acceptance occupied an important position in the present serial model. Greater perceived physical impact of MS was associated with lower illness acceptance, which was in turn associated with more psychological distress. Although illness acceptance was not independently associated with emotional overeating after psychological distress was included in the final regression model, the serial indirect association through illness acceptance and psychological distress was statistically supported. This pattern suggests that illness acceptance may be more closely related to emotional overeating through its association with psychological distress than through a direct relationship with eating behaviour. Previous research in MS has linked greater illness acceptance with more favourable health-related quality of life and psychological adjustment [11,12]. The present findings extend this perspective by indicating that illness acceptance may also be relevant when considering emotionally driven eating-related behaviours.

The disappearance of the direct association between perceived physical impact of MS and emotional overeating after accounting for illness acceptance and psychological distress is noteworthy. It indicates that subjective disease impact may be associated with emotional overeating primarily through psychological correlates of living with MS. This interpretation is broadly consistent with research by Plow et al. [1], who found that nutritional self-efficacy, physical activity, and communication with physicians were associated with healthier nutritional behaviours in people with MS, whereas impairment and activity limitation indicators were not retained as independent predictors in the final model. Although Plow et al. [1] examined healthy nutritional behaviours rather than emotional overeating, both studies support the view that psychosocial and self-management factors may be important for understanding eating-related behaviour in MS.

The adjusted inverse associations of disease duration and EDSS score with emotional overeating require particular caution. These associations were not the primary focus of the study and should not be interpreted as indicating that longer disease duration or greater neurological disability protects against emotional overeating. Instead, they may reflect unmeasured factors such as age, changes in appetite, mobility-related restrictions, altered daily routines, support from relatives, or practical limitations affecting food access and preparation. Previous studies of eating attitudes and disability in MS have produced inconsistent findings. Terzi et al. [36] found no association between disordered eating attitudes and EDSS score in a sample restricted to people with relapsing–remitting MS and relatively low disability. In contrast, İnanç et al. [37] observed a higher proportion of elevated Eating Attitudes Test scores among participants with EDSS scores of at least 4. These inconsistent findings may partly reflect differences in sample characteristics, disability range, and the eating-related constructs assessed. They also reinforce the need to avoid interpreting neurological disability as a straightforward determinant of emotional overeating.

The findings have potentially relevant implications for nutritional and multidisciplinary care in MS. Nutritional consultations frequently focus on dietary composition, nutritional deficiencies, body weight, and adherence to specific dietary patterns. These issues remain important, particularly because Mediterranean diet adherence has been associated with disability, quality of life, and depressive symptoms in MS populations [38]. However, the present findings suggest that discussions about food in clinical practice may also benefit from attention to current psychological distress, emotional triggers for eating, and adaptation to illness-related limitations. Assessment of psychological distress may be particularly relevant when patients report fluctuating eating habits, emotionally driven overeating, low illness acceptance, or difficulties maintaining health-related routines. Conversely, when elevated distress is identified, clinicians may consider asking about eating in response to emotional states as part of a broader psychosocial and nutritional assessment.

These implications should remain cautious. The present study did not assess dietary intake, body mass index, obesity, binge-eating symptoms, clinical eating disorders, or metabolic status. It also cannot establish whether changes in illness acceptance or psychological distress would be associated with changes in emotional overeating. Nevertheless, the results support an integrated approach in which nutritional counselling is complemented, when appropriate, by psychological assessment and interventions targeting distress, emotion regulation, and illness adjustment. Such multidisciplinary care may be particularly relevant when eating-related concerns co-occur with fatigue, sleep disturbances, pain, mood symptoms, or difficulties adapting to illness-related limitations.

### Strengths and Limitations

Several strengths of this study should be acknowledged. The analysis was conducted in a relatively large and clinically characterised sample of 382 people with MS, including participants with a broad range of disease duration and neurological disability. Neurological disability was assessed by a neurologist using the EDSS, while perceived disease impact, illness acceptance, psychological distress, and emotional overeating were assessed using established questionnaires with satisfactory to excellent internal consistency in the present sample. The theoretically informed model incorporated both subjective disease impact and psychological adjustment variables, while controlling for disease duration and neurological disability. Moreover, the overall pattern of indirect associations was retained in sensitivity analyses after additional adjustment for age and sex and when the total MSIS-29 score was substituted for the physical impact subscale. Finally, complete data were available for all included participants, avoiding the loss of information associated with missing-data procedures.

The findings should nevertheless be interpreted in light of several limitations. First, the cross-sectional design precludes conclusions about temporal ordering or causality. Although the serial indirect-association model was theoretically informed, alternative directions of association are possible; for example, emotional overeating may also contribute to psychological distress or be reciprocally related to it over time. Longitudinal studies with repeated assessments are needed to examine these possibilities. Furthermore, the ordering of illness acceptance and psychological distress was theory-informed but could not be empirically verified within a single cross-sectional assessment. Psychological distress may also be associated with lower illness acceptance, and reciprocal relationships are possible. Therefore, the serial indirect associations should be understood as a model-based decomposition of cross-sectional covariation rather than evidence of an intervening mechanism.

Second, the study used a convenience sample recruited through specialist clinical settings in Poland. Consequently, the findings may not generalise to all people with MS, particularly individuals treated exclusively outside specialist services or those living in different healthcare and cultural contexts. In addition, individuals with substantial MS-related cognitive difficulties and psychotic disorders were not eligible to participate; thus, the results should not be extrapolated to these patient groups. Although clinically apparent cognitive impairment was considered during eligibility screening, no formal neuropsychological assessment was performed. Therefore, the possible influence of milder cognitive difficulties on self-report questionnaire responses cannot be excluded.

Third, most variables were assessed by self-report, which may have introduced recall bias, response bias, and shared-method variance. Although the emotional overeating subscale of the MEH captures a tendency towards emotionally driven overeating, it does not establish the presence of binge-eating disorder, other clinical eating disorders, or objectively assessed dietary intake. Moreover, the psychometric performance of MEH Factor II was not examined separately in the present MS sample beyond internal consistency. The construct validity and measurement properties of this subscale require further evaluation specifically among people with MS. Detailed information on dietary quality, energy intake, body mass index, body composition, obesity, metabolic status, and nutritional status was not available for the present analysis. Medication-related factors were also not assessed in sufficient detail, including disease-modifying therapies, antidepressant use, corticosteroid treatment, and other medication classes that may influence appetite, mood, fatigue, body weight, or eating behaviour. Other potentially relevant factors, including fatigue, pain, sleep disturbances, physical activity, social support, and the home dietary environment, may also have influenced the observed associations.

Finally, the GHQ-12 was used as an indicator of current psychological distress rather than as a diagnostic measure of specific psychiatric disorders. Because several GHQ-12 items refer to sleep, concentration, everyday functioning, and perceived strain, some overlap with common MS-related symptoms such as fatigue, cognitive difficulties, and sleep disturbance cannot be excluded. Future studies should combine validated self-report instruments with clinical psychiatric assessment, prospective measures of eating behaviour and dietary intake, objective or clinician-rated assessments of fatigue and sleep disturbance, and more comprehensive clinical and biological data. In particular, longitudinal studies incorporating metabolic parameters, inflammatory markers, cortisol, medication profiles, and nutritional status would help clarify the biological and psychosocial pathways linking MS-related burden, psychological distress, and emotionally driven overeating. Such designs would also help determine whether changes in illness acceptance and psychological distress precede changes in emotional overeating and would provide a stronger basis for intervention development.

## 5. Conclusions

In this cross-sectional secondary analysis, greater perceived physical impact of MS was associated with higher emotional overeating; however, this association was no longer statistically significant after illness acceptance and psychological distress were considered. Psychological distress showed the strongest independent association with emotional overeating, and a statistically supported serial indirect association was observed through lower illness acceptance and greater psychological distress.

These findings indicate that emotional overeating in people with MS may be more closely related to psychological distress and illness adjustment than to perceived physical disease impact alone. Nutritional care in MS may therefore benefit from attention to emotional triggers for eating and, where appropriate, integration with psychological support targeting distress and adaptation to illness-related limitations. Longitudinal and intervention studies are required before implications for clinical practice can be established.

## Figures and Tables

**Table 1:** Sociodemographic and clinical characteristics of the sample (N = 382).

Characteristic	Value	Range/Note
Age, years	46.4 ± 11.9	18–82
Sex		
Female	256 (67.0%)	
Male	126 (33.0%)	
Marital status		
Married	257 (67.3%)	
Single	71 (18.6%)	
Divorced, separated, or widowed	54 (14.1%)	
Education level		
Primary or vocational education	96 (25.1%)	
Secondary education	165 (43.2%)	
Higher education	121 (31.7%)	
Employment status		
Employed	146 (38.2%)	
Disability pension	183 (47.9%)	
Retired	32 (8.4%)	
Unemployed	21 (5.5%)	
MS duration, years	11.5 ± 9.1	1–48
MS phenotype		
Relapsing–remitting MS	158 (41.4%)	
Secondary progressive MS	70 (18.3%)	
Primary progressive MS	88 (23.0%)	
Progressive–relapsing MS	33 (8.6%)	
Phenotype not reported	33 (8.6%)	Retained as recorded in the source database
EDSS score	4.35 ± 1.68	1.0–8.5

Note. Values are n (%) for categorical variables and mean ± SD for continuous variables unless otherwise indicated. MS phenotype coding follows the source database; the category “Phenotype not reported” was retained as recorded. EDSS, Expanded Disability Status Scale; MS, multiple sclerosis.

**Table 2:** Descriptive statistics and internal consistency of study variables.

No.	Variable	N	M	SD	Observed Range	Cronbach’s α
1	Emotional overeating (MEH Factor II)	382	4.03	2.78	0–10	0.80
2	MSIS-29 physical impact	382	43.48	18.41	20–100	0.98
3	Illness acceptance (AIS)	382	23.44	10.16	8–40	0.96
4	Psychological distress (GHQ-12)	382	27.91	5.14	12–42	0.81
5	EDSS	382	4.35	1.68	1.0–8.5	—
6	Disease duration, years	382	11.55	9.08	1–48	—

Note. Values are means and standard deviations unless otherwise indicated. Higher scores indicate greater emotional overeating, greater perceived physical impact of MS, greater illness acceptance, greater psychological distress, greater neurological disability, and longer disease duration. AIS, Acceptance of Illness Scale; EDSS, Expanded Disability Status Scale; GHQ-12, 12-item General Health Questionnaire; MEH, My Eating Habits Questionnaire; MSIS-29, Multiple Sclerosis Impact Scale-29.

**Table 3:** Pearson correlations among study variables.

No.	Variable	1	2	3	4	5	6
1	Emotional overeating	—					
2	MSIS-29 physical impact	0.22 ***	—				
3	Illness acceptance	−0.17 ***	−0.47 ***	—			
4	Psychological distress	0.39 ***	0.46 ***	−0.40 ***	—		
5	EDSS	−0.21 ***	0.11 *	−0.23 ***	−0.11 *	—	
6	Disease duration	−0.16 **	0.11 *	−0.27 ***	−0.01	0.48 ***	—

Note. Correlations are Pearson’s r. * *p* < 0.05; ** *p* < 0.01; *** *p* < 0.001. EDSS, Expanded Disability Status Scale; MSIS-29, Multiple Sclerosis Impact Scale-29.

**Table 4:** Hierarchical regression predicting emotional overeating.

**Panel A. Model summary**								
**Model**	R^2^	Adjusted R^2^	ΔR^2^	F	df	*p*	F Change	*p* Change
Model 1: Covariates	0.048	0.043	—	9.53	(2, 379)	<0.001	—	—
Model 2: +Physical impact	0.111	0.104	0.064	15.79	(3, 378)	<0.001	27.02	<0.001
Model 3: +Illness acceptance	0.133	0.123	0.021	14.40	(4, 377)	<0.001	9.18	0.003
Model 4: +Psychological distress	0.201	0.190	0.069	18.93	(5, 376)	<0.001	32.27	<0.001
**Panel B. Final model coefficients**								
**Predictor**	B	SE	Standardized β	*t*	*p*	95% CI Lower	95% CI Upper	
Disease duration	−0.038	0.016	−0.123	−2.30	0.022	−0.070	−0.005	
EDSS	−0.228	0.089	−0.138	−2.56	0.011	−0.404	−0.053	
MSIS-29 physical impact	0.010	0.008	0.069	1.24	0.216	−0.006	0.027	
Illness acceptance	−0.023	0.015	−0.083	−1.47	0.143	−0.053	0.008	
Psychological distress	0.169	0.030	0.312	5.68	<0.001	0.110	0.227	

Note. Model 1 included disease duration and EDSS. Model 2 added perceived physical impact of MS. Model 3 added illness acceptance. Model 4 added psychological distress. The final model explained 20.1% of the variance in emotional overeating. All VIF values in the final model were ≤1.50. EDSS, Expanded Disability Status Scale; MSIS-29, Multiple Sclerosis Impact Scale-29.

**Table 5:** Serial indirect associations between perceived physical impact of MS and emotional overeating.

Panel A. Regression paths						
Path	B	SE	t	*p*	95% CI Lower	95% CI Upper
a1: Physical impact → Illness acceptance	−0.245	0.024	−10.10	<0.001	−0.293	−0.197
a2: Physical impact → Psychological distress	0.097	0.014	7.11	<0.001	0.070	0.124
d21: Illness acceptance → Psychological distress	−0.143	0.026	−5.56	<0.001	−0.194	−0.093
b1: Illness acceptance → Emotional overeating	−0.023	0.015	−1.47	0.143	−0.053	0.008
b2: Psychological distress → Emotional overeating	0.169	0.030	5.68	<0.001	0.110	0.227
c’: Direct physical impact → Emotional overeating	0.010	0.008	1.24	0.216	−0.006	0.027
c: Total physical impact → Emotional overeating	0.038	0.007	5.20	<0.001	0.024	0.053
*Panel B. Bootstrap Indirect Associations (5000 Percentile Resamples)*						
**Indirect association**			**Estimate,** **B**	**95% CI** **Lower**	**95% CI** **Upper**	**Statistically** **Supported**
Via illness acceptance only			0.006	−0.002	0.013	No
Via psychological distress only			0.016	0.009	0.025	Yes
Sequential: physical impact → illness acceptance → psychological distress → emotional overeating			0.006	0.003	0.009	Yes
Total indirect association			0.028	0.017	0.039	Yes

Note. Unstandardised regression coefficients are reported. Standardized path coefficients presented in the text and Figure 1 were derived from separately fitted regression equations using z-standardized variables and identical model specifications. The primary serial indirect-association model specified perceived physical impact of MS (MSIS-29 physical impact) as the independent variable and emotional overeating as the dependent variable, with illness acceptance and psychological distress entered as serially ordered variables. Disease duration and EDSS were included as covariates in all regression equations. The specified order was theory-informed and does not imply temporal or causal precedence. A 95% bootstrap confidence interval excluding zero indicates a statistically supported indirect association. EDSS, Expanded Disability Status Scale; MSIS-29, Multiple Sclerosis Impact Scale-29.

## Data Availability

The data presented in this study are available on reasonable request from the corresponding author.

## Supplementary Materials

The following supporting information can be downloaded at: https://www.mdpi.com/article/10.3390/nu18162642/s1, Table S1: Regression Diagnostics for the Final Hierarchical Regression Model Predicting Emotional Overeating. Table S2: Sensitivity Analyses for the Serial Indirect-Association Model Linking Perceived Multiple Sclerosis Impact with Emotional Overeating.

## References

[1] Plow M., Finlayson M., Cho C. (2012). Correlates of nutritional behavior in individuals with multiple sclerosis. Disabil. Health J..

[2] Riemann-Lorenz K., Eilers M., von Geldern G., Schulz K.-H., Köpke S., Heesen C. (2016). Dietary interventions in multiple sclerosis: Development and pilot-testing of an evidence-based patient education program. PLoS ONE.

[3] Reichenberger J., Schnepper R., Arend A.-K., Blechert J. (2020). Emotional eating in healthy individuals and patients with an eating disorder: Evidence from psychometric, experimental and naturalistic studies. Proc. Nutr. Soc..

[4] Warren A., Frame L.A. (2025). Restoring a healthy relationship with food by decoupling stress and eating: A translational review of nutrition and mental health. Nutrients.

[5] Konttinen H. (2020). Emotional eating and obesity in adults: The role of depression, sleep and genes. Proc. Nutr. Soc..

[6] Dakanalis A., Mentzelou M., Papadopoulou S.K., Papandreou D., Spanoudaki M., Vasios G.K., Pavlidou E., Mantzorou M., Giaginis C. (2023). The association of emotional eating with overweight/obesity, depression, anxiety/stress, and dietary patterns: A review of the current clinical evidence. Nutrients.

[7] Ha O.R., Lim S.L. (2023). The role of emotion in eating behavior and decisions. Front Psychol..

[8] Thompson A.J., Baranzini S.E., Geurts J., Hemmer B., Ciccarelli O. (2018). Multiple sclerosis. Lancet.

[9] Dennison L., Moss-Morris R., Chalder T. (2009). A review of psychological correlates of adjustment in patients with multiple sclerosis. Clin. Psychol. Rev..

[10] Wilski M., Brola W., Łuniewska M., Tomczak M. (2021). The perceived impact of multiple sclerosis and self-management: The mediating role of coping strategies. PLoS ONE.

[11] Pakenham K.I., Fleming M. (2011). Relations between acceptance of multiple sclerosis and positive and negative adjustments. Psychol. Health.

[12] Wilski M., Gabryelski J., Brola W., Tasiemski T. (2019). Health-related quality of life in multiple sclerosis: Links to acceptance, coping strategies and disease severity. Disabil. Health J..

[13] Boeschoten R.E., Braamse A.M.J., Beekman A.T.F., Cuijpers P., van Oppen P., Dekker J., Uitdehaag B.M. (2017). Prevalence of depression and anxiety in multiple sclerosis: A systematic review and meta-analysis. J. Neurol. Sci..

[14] Polman C.H., Reingold S.C., Banwell B., Clanet M., Cohen J.A., Filippi M., Fujihara K., Havrdova E., Hutchinson M., Kappos L. (2011). Diagnostic criteria for multiple sclerosis: 2010 revisions to the McDonald criteria. Ann. Neurol..

[15] Ogińska-Bulik N., Putyński L. (2000). Kwestionariusz Moje Zwyczaje Żywieniowe—Konstrukcja i własności psychometryczne. Acta Univ. Lodz. Folia Psychol..

[16] Bartosiewicz A., Dereń K., Łuszczki E., Zielińska M., Nowak J., Lewandowska A., Sulikowski P. (2025). Exploring body composition and eating habits among nurses in Poland. Nutrients.

[17] Jaruga-Sękowska S., Staśkiewicz-Bartecka W., Woźniak-Holecka J. (2025). The impact of social media on eating disorder risk and self-esteem among adolescents and young adults: A psychosocial analysis in individuals aged 16–25. Nutrients.

[18] Hobart J., Lamping D.L., Fitzpatrick R., Riazi A., Thompson A.J. (2001). The multiple sclerosis impact scale (MSIS-29): A new patient-based outcome measure. Brain.

[19] Jamroz-Wiśniewska A., Papuć E., Bartosik-Psujek H., Belniak E., Mitosek-Szewczyk K., Stelmasiak Z. (2007). Validation of selected aspects of psychometry of the Polish version of the Multiple Sclerosis Impact Scale 29 (MSIS-29). Neurol. Neurochir. Pol..

[20] Riazi A., Hobart J.C., Lamping D.L., Fitzpatrick R., Thompson A.J. (2003). Evidence-based measurement in multiple sclerosis: The psychometric properties of the physical and psychological dimensions of three quality of life rating scales. Mult. Scler..

[21] Hoogervorst E.L.J., Zwemmer J.N., Jelles B., Polman C.H., Uitdehaag B.M.J. (2004). Multiple Sclerosis Impact Scale (MSIS-29): Relation to established measures of impairment and disability. Mult. Scler..

[22] Felton B.J., Revenson T.A., Hinrichsen G.A. (1984). Stress and coping in the explanation of psychological adjustment among chronically ill adults. Soc. Sci. Med..

[23] Juczyński Z. (2001). Narzędzia Pomiaru W Promocji I Psychologii Zdrowia.

[24] Cepuch G., Oskędra I., Olma M., Wojtas K. (2016). The quality of life with regard to the sexual sphere and acceptance of the disease in young adults suffering from multiple sclerosis. Przegl Lek..

[25] Goldberg D., Williams P. (1988). A User’s Guide to the General Health Questionnaire.

[26] Makowska Z., Merecz D., Mościcka A., Kolasa W. (2002). The validity of General Health Questionnaires, GHQ-12 and GHQ-28, in mental health studies of working people. Int. J. Occup. Med. Env. Health.

[27] Gnambs T., Staufenbiel T. (2018). The structure of the General Health Questionnaire (GHQ-12): Two meta-analytic factor analyses. Health Psychol. Rev..

[28] Rabins P.V., Brooks B.R. (1981). Emotional disturbance in multiple sclerosis patients: Validity of the General Health Questionnaire. Psychol. Med..

[29] Nicholl C.R., Lincoln N.B., Francis V.M., Stephan T.F. (2001). Assessment of emotional problems in people with multiple sclerosis. Clin. Rehabil..

[30] Kurtzke J.F. (1983). Rating neurologic impairment in multiple sclerosis: An expanded disability status scale (EDSS). Neurology.

[31] Uitdehaag B.M.J. (2018). Disability outcome measures in phase III clinical trials in multiple sclerosis. CNS Drugs.

[32] Hayes A.F. (2022). Introduction to Mediation, Moderation, and Conditional Process Analysis: A Regression-Based Approach.

[33] Kara B., Çelik A. (2015). The relationship between risk for eating disorder and health-related quality of life in patients with multiple sclerosis. Gulhane Med. J..

[34] Saadat S., Hosseininezhad M. (2021). Comparison of eating behaviors in people with multiple sclerosis with recurrence in the past year and control group. J. Shahid Sadoughi Univ. Med. Sci..

[35] Dehghan M., Sharifi P., Hasani J., Young C.A., Langdon D. (2023). Healthier living with MS: The key role of self-efficacy and emotion regulation. Mult. Scler. Relat. Disord..

[36] Terzi M., Kocamanoğlu B., Güz H., Onar M. (2009). The eating attitudes in multiple sclerosis patients. J. Neurol. Sci..

[37] İnanç Y., Turgut C., Kaya T. (2023). Eating attitude in multiple sclerosis. KSU Med. J..

[38] Dakanalis A., Tryfonos C., Pavlidou E., Vadikolias K., Papadopoulou S.K., Alexatou O., Vorvolakos T., Chrysafi M., Fotiou D., Mentzelou M. (2024). Associations between Mediterranean diet adherence, quality of life, and mental health in patients with multiple sclerosis: A cross-sectional study. J. Pers. Med..

