# Supplementary material for: Psychological Distress Is Associated with Emotional Overeating in Multiple Sclerosis: The Role of Perceived Disease Impact and Illness Acceptance—A Cross-Sectional Study
Source: Nutrients. 2026 Aug 12;18(16):2642. doi: 10.3390/nu18162642 (PMC13516604; doi:10.3390/nu18162642)
Supplement: Supplementary file 1 [file nutrients-18-02642-s001.zip › nutrients-4442351-supplementary.pdf]

Supplementary Materials

Table S1. Regression Diagnostics for the Final Hierarchical Regression Model Predicting Emotional Overeating.

Diagnostic	Result	Interpretation
Variance inflation factor (VIF)	Range: 1.35-1.50	No meaningful multicollinearity
Maximum Cook's distance	0.034	No single observation exerted undue influence
Durbin-Watson statistic	1.90	Reported for completeness; Near 2.0; reported for descriptive completeness

Note. Diagnostics refer to the final hierarchical regression model including disease duration, Expanded Disability Status Scale score, perceived physical impact of multiple sclerosis, illness acceptance, and psychological distress. Multicollinearity was assessed using variance inflation factors, and influential observations were evaluated using Cook's distance. Residual plots and normal Q–Q plots were visually inspected to assess linearity, homoscedasticity, and normality of residuals. The Durbin–Watson statistic is reported for descriptive completeness; residual autocorrelation is not a primary concern in cross-sectional data. VIF, variance inflation factor.

Table S2. Sensitivity Analyses for the Serial Indirect-Association Model Linking Perceived Multiple Sclerosis Impact with Emotional Overeating.

Model Specification	Total Indirect Association, B	95% Bootstrap CI	Sequential Indirect Association, B	95% Bootstrap CI
Primary model: physical MS impact; adjusted for disease duration and EDSS	0.028	0.017 to 0.039	0.006	0.003 to 0.009
Primary model with additional adjustment for sex and age	0.028	0.017 to 0.040	0.006	0.003 to 0.009
Alternative model: total MSIS-29 score substituted for physical MS impact	0.018	0.010 to 0.027	0.003	0.001 to 0.005

Note. All models were estimated using 5,000 percentile bootstrap resamples. The serial indirect association represents perceived MS impact → illness acceptance → Psychological distress → emotional overeating. An indirect association was considered statistically supported when the 95% bootstrap confidence interval did not include zero. B values are unstandardised indirect-association estimates. EDSS, Expanded Disability Status Scale; MS, multiple sclerosis; MSIS-29, Multiple Sclerosis Impact Scale-29.
